# Quantum Buckling in Metal–Organic Framework Materials

**DOI:** 10.1021/acs.nanolett.1c03579

**Published:** 2021-12-09

**Authors:** R. Matthias Geilhufe

**Affiliations:** Nordita, KTH Royal Institute of Technology and Stockholm University, Roslagstullsbacken 23, 10691 Stockholm, Sweden

**Keywords:** Phase transition, Quantum materials, Metal−organic frameworks, Quantum buckling, Bucklon, Transverse field Ising model

## Abstract

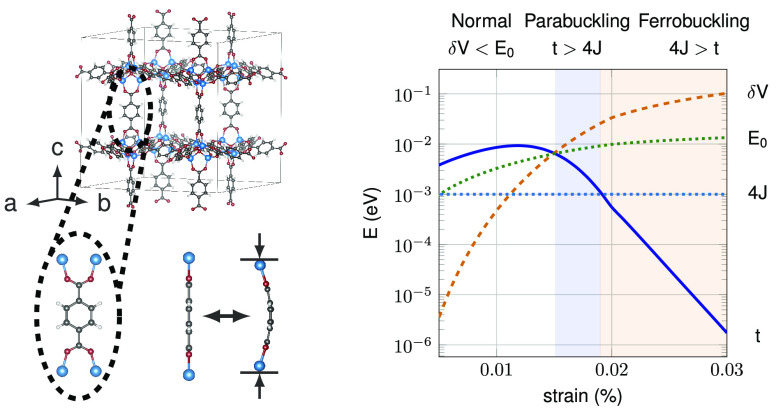

Metal–organic frameworks are porous materials composed of metal ions or clusters coordinated by organic molecules. As a response to applied uniaxial pressure, molecules with a straight shape in the framework start to buckle. At sufficiently low temperatures, this buckling has a quantum nature described by a superposition of degenerate buckling states. Buckling states of adjacent molecules couple in a transverse field Ising type behavior. Based on the example of the metal organic framework topology MOF-5, we derived the phase diagram under applied strain, showing a normal phase, a parabuckling phase, and a ferrobuckling phase. At zero temperature, quantum phase transitions between the three phases can be induced by strain. This novel type of order opens a new path toward strain induced quantum phases.

Under sufficient axial load, a column responds by a sudden deformation, namely buckling. The deformation corresponds to a classical solution minimizing the action. At the nanometer scale, the electrostatic control of buckling was recently realized, giving rise to buckling bits for nanomechanical computation.^1^ Quantum effects become dominant when the column size is decreased even further, allowing for tunneling between degenerate buckling states. Recently, this line of thought has initiated research to realize mechanical qubits.^2,3^ Prominent designs has been proposed, such as those based on carbon nanotubes.^4^ Realizing the entanglement between various adjacent mechanical qubits has remained an open question.

Metal–organic framework materials are compounds built of metal ions or clusters coordinated by organic ligands. After the first MOFs were realized in the late 1990s,^5^ more than 90 000 stable structures have been synthesized and characterized to date.^6^ They have been intensively discussed in the context of gas sorption and storage, catalysis, electronic devices, etc.^7−9^ Mechanical properties and the flexibility of MOFs are summarized in ref (10).

The present paper shows how uniaxial pressure in MOFs can be used to induce the quantum buckling of ligand molecules. Interestingly, the buckling of individual molecules is not independent but instead interacts similarly to a transverse field Ising model. We motivate the model on the example of molecular buckling in the system MOF-5. Depending on the applied uniaxial pressure, the system undergoes two quantum phase transitions, first from a normal phase to a parabuckling phase and second from a parabuckling phase to a ferrobuckling phase. In the parabuckling phase, elementary excitations exhibit a gap (mass) of . At the parabuckling-ferrobuckling phase transition (*t* = 4*J*), the gap closes. The parabuckling–ferrobuckling quantum critical point might give rise to novel types of fluctuation-induced order, such as strain-controlled superconductivity.

MOF-5 describes a cubic framework topology^5^ with the sum formula Zn_4_O(BDC)_3_. BDC is an abbreviation for 1,4-benzenedicarboxylate (Figure 1a). The structure is open, with a spacing of 12.94 Å between the centers of adjacent Zn_4_O clusters. MOF-5 is extremely soft, with a bulk modulus of 15.37 GPa.^11^ Applying strain along one of the Cartesian axes, the bond lengths within the Zn_4_O clusters and BDC molecules are squeezed up to the point where the structure responds by a buckling of the molecules. To approximate the effect, we monitored the change in the total energy upon buckling for individual BDC molecules bound to Zn atoms and applied strain along the Cartesian *z*-direction. Calculations based on density functional theory were performed using VASP.^12^ The exchange-correlation functional was approximated by the MetaGGA functional SCAN program^13^ in combination with the rVV10 van der Waals corrections.^14,15^ The energy cutoff was set to 700 eV. Zn d-electrons were treated with a Hubbard-U correction of 2 eV.^16^

**Figure 1:**
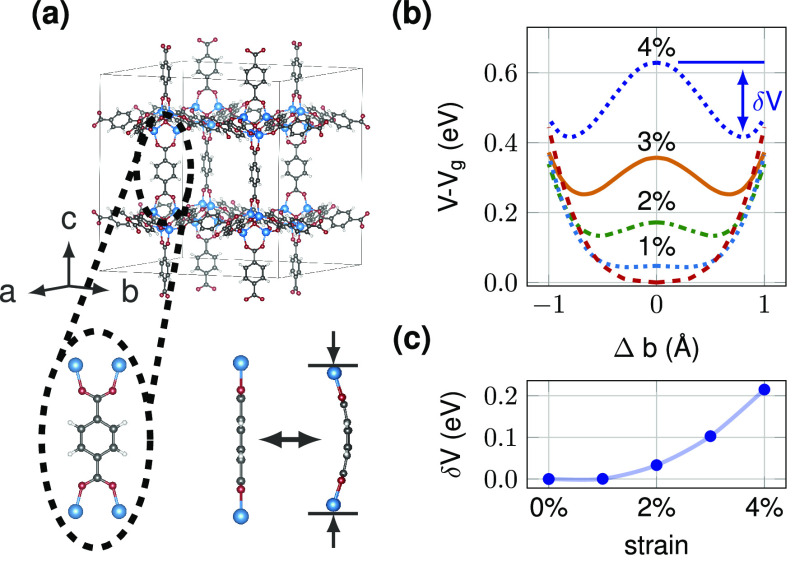
Buckling of BDC molecules in MOF-5. (a) The crystal structure of MOF-5. Zn_4_O clusters are coordinated by BDC molecules, forming a periodic 3D cubic network. BDC molecules can be buckled by uniaxial pressure. (b) Total energy *V* versus the buckling of BDC molecules for various strain values. *V*_*g*_ denotes the equilibrium total energy. Under strain, the total energy follows the form of a double-well potential. (c) Barrier height δ*V* of the double-well potential against uniaxial strain along the Cartesian *z*-axis.

The buckling potential for various strain strengths is shown in Figure 1b. By applying strain, the total energy increases, and the energy profile forms a double-well potential. The potential has two degenerate minima, the left- and right-buckled state. To the lowest order, the potential can be described by a fourth-order polynomial
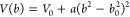
1where *a* is an overall scaling factor, *b*_0_ is the potential minimum or classical solution, and *V*_0_ is the characteristic energy. It coincides with the potential minimum for the zero-strain case (*b*_0_ = 0). Note that all three parameters are strain-dependent (see Table 1).

**Table 1:** Fitting Parameters for the Double-Well Potential^a^

	*V*_0_ (eV)	*a* (eV Å^–4^)	*b*_0_ (Å)
1%	–171.70	0.35	0.19
2%	–171.61	0.40	0.54
3%	–171.49	0.45	0.69
4%	–171.33	0.51	0.81

a The table shows the strain dependence of the characteristic energy *V*_0_, the scaling factor *a*, and the classical solution or potential minimum *b*_0_.

In the following, we derive the quantum buckling Hamiltonian of strained MOF-5. First, assume a sufficiently high potential barrier. Then, focus on one potential well, e.g., the right-buckled solution. The corresponding energy is *E*_g_ and can be approximated as follows. Around the classical solution *b* ≈ *b*_0_, the potential is harmonic with , i.e., a quantum harmonic oscillator. The ground state energy is given by
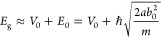
2

The wave function ψ_*r*_ is normalized as |ψ_r_|^2^ = 1 over the right potential well. A similar construction is done for the wave function ψ_l_ describing the buckling in the left potential well. As the barrier between both wells is finite, tunneling is allowed. We describe the effective Hamiltonian incorporating the tunneling with tunneling strength *t* by
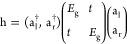
3

Here, a_l_^†^ and a_r_^†^ (a_*l*_ and a_*r*_) are the creation (annihilation) operators for the left-buckled and right-buckled solution, respectively. The energy levels correspond to the symmetric and antisymmetric solutions of the system
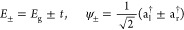
4

Following ref (2), we can approximate *t* as follows:
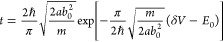
5

So far, we have focused on the buckling of a single molecule. However, MOF-5 is a lattice periodic framework. As a result, the buckling of adjacent molecules *i* and *j* is coupled, with the coupling strength *J*_*ij*_. As shown in Figure 2, for *J*_*ij*_ > 0 (*J*_*ij*_ < 0) the buckling in the same (opposite) buckling state is energetically preferred. Merging this result with the single-molecule buckling Hamiltonian of eq 3, we can write down the effective quantum buckling Hamiltonian for the MOF in the following way (we neglect the constant energy shift *E*_0_):
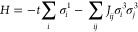
6

**Figure 2:**
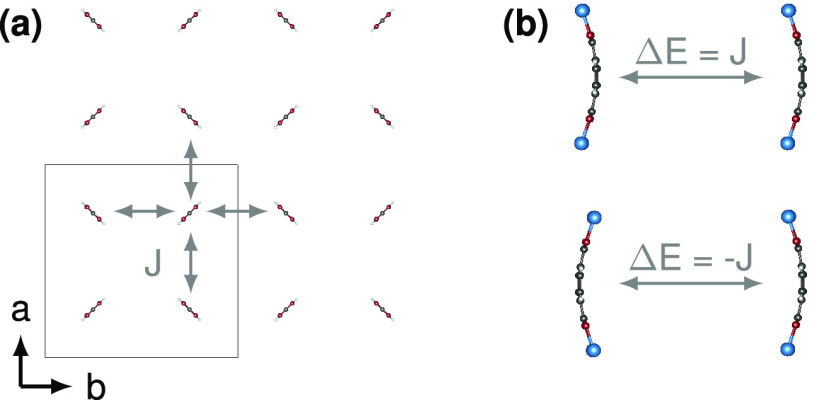
Exchange interaction of buckled linker molecules. (a) The molecular arrangement of BDC linker molecules in MOF-5 in the ***a***–***b*** plane. Molecules form the pattern of a square lattice. (b) The energy difference between ferro- and antiferrobuckling.

Equation 6 is the mechanical buckling version of the well-studied transverse field Ising model. The transverse field Ising model has been applied intensively to study order–disorder ferroelectrics, simple ferromagnets, simple Jahn–Teller systems, and more.^17,18^ In the following, we summarize a few key results and their interpretation for quantum buckling.

The zero-temperature phase diagram of strained MOF-5 is shown in Figure 3. Depending on the strain strength, three phases are present. For sufficiently weak strain, the double-well potential barrier is lower than the lowest-lying level of the single-well solution (δ*V* < *E*_0_). As a result, the quantum state of the molecule does not experience the presence of two distinct minima, and the material remains in a normal state with no buckling present. On the opposite side , for sufficiently large strain and dominating exchange  the MOF will show ordered buckling. The simplest order possible would be the ferrobuckling state (*J*_*ij*_ > 0), with all molecules occupying buckling states that point in the same direction, i.e.,  and . Between the normal and the ferrobuckling phase is the parabuckling phase. Here, the energy of the single-well solutions lies below the potential barrier (δ*V* > *E*_0_). Additionally, the tunneling term for each molecule dominates the exchange between adjacent molecules (*t* > 4*J*), i.e., and .

**Figure 3:**
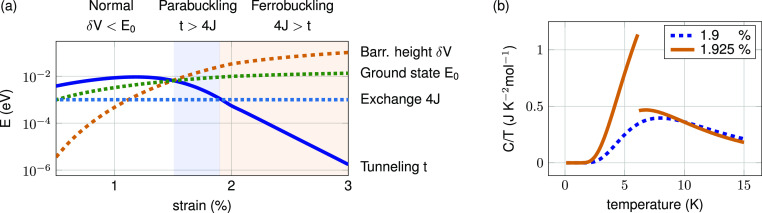
(a) Phase diagram of strained MOF-5. If the single-well ground state energy is higher than the barrier height, no buckling occurs (normal phase). For a large enough barrier height and dominating tunneling *t* > 4*J*, molecules are in a superposition of left and right buckled states with no long-range order (parabuckling). For the dominating exchange energy 4*J* > *t*, buckled molecules form long-range order. For MOF-5, the expected transition temperature for 3% strain is ≈10 K. (b) Specific heat close to the quantum phase transition between para- and ferrobuckling. In the ferrobuckling regime, the specific heat contribution of the collective buckling state exhibits a discontinuity at the transition temperature.

In the mean-field approximation, we write eq 6 in terms of single-site contributions as follows:^18^
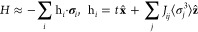
7

Assuming weak variations in the buckling state  and the nearest neighbor approximation, each single-site contribution **h**_*i*_·**σ**_*i*_ has eigenvalues . As a result, we obtain  for the buckling per site, where . From the expectation value , we can deduce the critical temperature given by
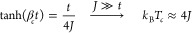
8

To estimate the exchange *J* for MOF-5, we performed DFT total energy calculations on a pair of molecules for the two configurations shown in Figure 2. The exchange *J* was then obtained from the energy difference of the ferro- and antiferrobuckled configurations. The result was a fairly constant value of ≈0.25 meV in the low-strain regime. Hence, according to eq 8, the transition temperature is ≈10 K. We note that we focus on a regime of sufficiently small strain, which justifies the nearest neighbor approximation. A more detailed investigation of the strength of the nearest neighbor, second-nearest neighbor, and 4σ^3^ interactions would be necessary but is outside the scope of this paper. In particular, the latter is expected to become significant for large strain. Such an extended four-spin transverse field Ising model is in close connection to the eight-vertex model, which has been intensively discussed.^19,20^ Such a model also captures the transition between ordered and glass states.

Besides the transition temperature, the mean-field approximation allows us to estimate the ensemble average of the enegry, given by . We numerically evaluate the specific heat contribution due to collective buckling as , taking into account the implicit temperature dependence of . We plotted *C*/*T* per mole, as shown in Figure 3b, for a strain close to the quantum critical point between para- and ferrobuckling. In the parabuckling regime (dotted blue line, 1.9% strain) the specific heat is a a smooth function in temperature. In contrast, in the ferrobuckling regime (solid orange line, 1.925% strain), a phase transition at a temperature of ≈6.15 K leads to a discontinuity in the specific heat. Hence, a strain-dependent measurement of the specific heat at low temperatures could provide experimental evidence for the quantumbuckling phases.

Collective excitations of the ordered buckling states emerge similarly to magnons in magnetically ordered systems.

The bucklon excitations of the parabuckling phase can be estimated by evaluating the Heisenberg equation of motion to calculate . Using the random phase approximation ,^18^ we obtain for the effective square lattice of MOF-5
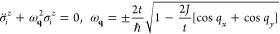
9

At ***q*** = 0, the spectrum has a gap . As a result, interactions mediated by bucklons in the parabuckling phase are exponentially decaying and short-ranged. At the quantum phase transition between the ferrobucking phase and the parabuckling phase *t* ≈ 4*J*, the gap closes, leading to significantly enhanced interactions due to strong fluctuations.

For example, fluctuations at the quantum critical point between the para and ferroelectric phase in quantum ferroelectrics enhance the superconductivity.^21,22^ In contrast to their ferroelectric counterparts, the quantum parabuckling–ferrobuckling phase transition can be induced straightforwardly by uniaxial pressure (the calculated bulk modulus of MOF-5 is 15.37 GPa,^11^ whereas the bulk modulus of SrTiO_3_ is 172.1 GPa^23^). Modifying the formalism of Edge et al.^22^ and the strong-coupling theory of McMillan^24^ to the present case of electron–bucklon-mediated coupling, the superconducting coupling strength goes as
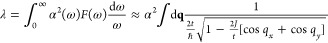
10

Here, we assumed a constant coupling constant α^2^(ω) ≈ α^2^ and a spectral density *F*(ω) = *∫*d***q*** δ(ω – ω_***q***_). Hence, at the ferrobuckling transition where  and ω_***q***=0_ → 0, the superconducting interaction strength is significantly enhanced. We note that MOF-5 is an insulator with a gap of ≈3.4 eV.^11^ Hence, to observe the superconductivity, the material would have to be doped accordingly. Currently, only very few examples of superconductivity in MOFs are known.^25,26^ These examples are most likely based on strong correlations, similar to high *T*_c_ superconductors. We believe the emergence of order due to quantum critical buckling is a different and a more general concept in MOFs. This concept goes beyond MOF-5 and superconductivity.

In summary, we showed that applying uniaxial pressure to MOFs can lead to the buckling of the organic linker molecules. At low temperatures, the buckling of individual molecules is of a quantum nature, where the molecule is in a superposition of left- and right-buckled states. Additionally, the buckling of adjacent molecules is not independent but instead weakly coupled. As a result, the material can undergo a phase transition into a collective para- and ferrobuckling state. MOFs are soft. Therefore, the tuning of the quantum buckling phases by uniaxial pressure can be achieved straightforwardly. The emergence of a phase transition at low temperatures should be seen in the specific heat of the material. For MOF-5 and a strain of ≈2–5%, we expect the phase transition into a ferrobuckling phase to take place at ≈10 K. By slowly decreasing the uniaxial pressure to <2%, a quantum phase transition to the parabuckling phase is expected. The quantum critical point between the two phases might be a prominent experimental platform to investigate novel types of fluctuation-induced order.
